# Amaranth leaf extract protects against hydrogen peroxide induced oxidative stress in *Drosophila melanogaster*

**DOI:** 10.1186/s13104-021-05603-x

**Published:** 2021-05-17

**Authors:** Ndinawe Johnmark, Hellen W. Kinyi

**Affiliations:** 1 School of Pharmacy, Kampala International University, Kampala, Uganda; 2 Department of Biochemistry, School of Medicine, Kabale University, Kabale, Uganda

**Keywords:** Oxidative stress, Amaranths, *Drosophila melanogaster*, Antioxidant*s*

## Abstract

**Objective:**

Amaranths leaves are rich in ascorbic acid and polyphenol compounds which have antioxidant activity. The aim of this study was to evaluate their in vivo antioxidant activity. The effect of consumption of Amaranth leaf extract on in vivo antioxidant activity, catalase enzyme activity and H_2_O_2_ induced oxidative stress in *Drosophila melanogaster* flies was assessed.

**Results:**

Consumption of Amaranth leaf extract was associated with increased survival on exposure to H_2_o_2_ in a dose dependent manner in *Drosophila melanogaste*r flies. The study concludes that the ethanolic extract of Amaranth leaves offer protection against hydrogen peroxide-induced oxidative stress.

**Supplementary Information:**

The online version contains supplementary material available at 10.1186/s13104-021-05603-x.

## Introduction

Oxidative stress refers to an imbalance arising due to excess production of free radicals and reduced activity of antioxidants. Free radicals are atoms or molecules that contain unpaired electrons and react with other molecules by taking or giving electrons [1]. They can be products of normal cellular metabolism or environmental stressors and have been implicated in the pathogenesis of many diseases [2, 3].

Antioxidant compounds neutralize harmful effects of free radicals by either preventing their formation or removing them before they can cause damage to cellular structures [4]. Consumption of fruits and vegetables has been linked to reduction in the incidence of oxidative-stress related diseases such as; cancer, diabetes, neurodegenerative diseases, inflammation as well as cell and cutaneous aging issues [5]. Determination of the in vivo antioxidant activity of fruits and vegetables provides a scientific basis to justify the hypothesis that consumption of these plant species offers protection against oxidative-stress related diseases.

Amaranths, the generic name by which the vegetables of the genus *Amaranthus* are referred to are widely distributed short-lived herbs that occur in temperate and tropical regions. The *Amaranthus* species is one of the few plants from which leaves are eaten as a vegetable while the seeds are eaten as cereals [6]. The in vitro antioxidant activity of Amaranths leaves has been well documented and attributed to both vitamin C and polyphenol compounds found in extracts [7–9]. The specific objective of this work was to evaluate the in vivo protective activity of Amaranths against hydrogen peroxide induced oxidative stress in male *Drosophila melanogaster.* Male W1118 (white) strain flies have been chosen as they are more susceptible to oxidative stress [10, 11] and hence the consumption of Amaranth leaves is likely to rescue the lethality of hydrogen peroxide.

## Methods

### Preparation of experimental food

Amaranth leaves used in the study were purchased from a vendor in the Central market of Ishaka town of Bushenyi Municipality of Western Uganda, were taxonomically identified at Mbarara University by Dr. Apio Opolot and voucher specimens were given voucher number HWK 001 *Amaranthus dubius*. Extraction was done as previously described [12] (Details, see Additional file 1). To prepare the fly food with Amaranth and ascorbic acid, 0.25 mg and 0.5 mg of Amaranth extract and ascorbic acid were each dissolved in 10mls of distilled water to obtain a concentration of 2.5 mg/ml and 5 mg/ml respectively. Each was separately added to 490mls of molten fly food and mixed thoroughly to obtain a mixture with Amaranth extract and Ascorbic acid each at a concentration of 0.05 mg/ml and 0.1 mg/ml. These mixtures were then poured in labeled vials and left to cool. The food was covered with cotton balls and stored at 4 ℃.

### In vitro antioxidant activity

The in vitro antioxidant activity of the Amaranth leaf extract was determined using the 2, 2-diphenyl-1-picrylhydrazyl (DPPH) radical radical-scavenging assay [13] (Details, see Additional file 1).

### Flies and fly handling

The model used for the study was the *w1118* (white) strain of *Drosophila melanogaster*, a gift from Dr. Isabel Palacios (UK) and maintained at the Institute of Biomedical Research, Kampala International University Western Campus, Ishaka-Bushenyi, Uganda. They were fed on cornmeal-yeast-agar medium [14] and maintained at 25 ℃ under a 12/12 h light and dark cycle in a digital fly incubator. From the stock population, virgin females and young males were placed on fresh food and allowed to mate. Fresh eggs were collected, and dated to ensure that the dates of birth are synchronized for all flies. From these, 450 male adult flies were collected and divided into 3 groups to be fed on: normal food (NF), Amaranth extract, and ascorbic acid (ASA) groups. The NF (90 flies), was fed on the standard cornmeal diet, the Amaranth group (180 flies) fed on the Amaranth-fly food mixture and ASA group (180 flies) on ASA-fly food mixture. To determine the in vivo antioxidant activity, catalase enzyme activity and resistance to oxidative stress, groups of *Drosophila* flies were fed on the respective diets for 5 days [15].

### Determination of in vivo antioxidant activity

Ten flies each from the NF, Amaranth and ASA groups were separately anaesthetized on ice and homogenized in 100 µl of cold 0.05% phosphate buffered saline tween (PBST) solution (pH 7.4). The homogenate was centrifuged at 4000 g for 10 min and the supernatant collected and used for the determination of 2, 2-diphenyl-1-picrylhydrazyl radical (DPPH) scavenging activity. DPPH scavenging activity was determined as previously described [13]. Briefly, 50 μl of the fly homogenate was added to 5 mls of 0.004% methanol solution of DPPH. This mixture was incubated in the dark at room temperature for 30 min. The absorbance of the mixture was then read at 517 nm against a DPPH blank. The assay was carried out in triplicate and percentage of inhibition was calculated using the formula:

$$ \% {\text{ Inhibition}}\, = \,\left[ {\left( {{\text{AB}} - {\text{AA}}} \right)/{\text{AB}}} \right]^{*} {1}00 $$

where AB = Absorbance of blank; AA = Absorbance of test.

### Determination of resistance to hydrogen peroxide induced oxidative stress

The resistance to hydrogen peroxide-induced oxidative stress was conducted as previously described with minor modifications [16]. Briefly, ten fruit flies from each of the NF, Amaranth and ASA groups were transferred to empty vials after 5 days of feeding. The flies were starved for 6 h to stimulate the uptake of hydrogen peroxide. Afterwards, the flies were transferred to vials containing only filter paper soaked with 1% hydrogen peroxide in 5% sucrose solution. The soaked filter paper was changed daily and flies alive/dead were recorded every day until the last one died.

### Catalase enzyme activity

The assay was carried out on *Drosophila* flies fed on the NF, Amaranth and ASA food for five days. Ten flies per group anaesthetized by chilling on ice were homogenized in ice cold phosphate buffer saline (pH 7.4) at a weight: buffer ratio of 1:10 and centrifuged at 2500 rpm for 10 min. The resulting supernatant was collected and used for the catalase assay. The catalase activity was measured as previously described [17]. (Details see Additional file 1**)**.

### Statistical analysis

Data was analyzed using the free software Paleontological statistics software (PAST 3), expressed as mean and standard deviation and presented as graphs. Data of DPPH scavenging and catalase activities were analyzed using factorial analysis of variance (ANOVA) followed by a Tukey’s multiple comparison test. Oxidative stress resistance was determined using Kaplan–Meier survival analysis with significance set at P < 0.05.

## Results

**Fig. 1:**
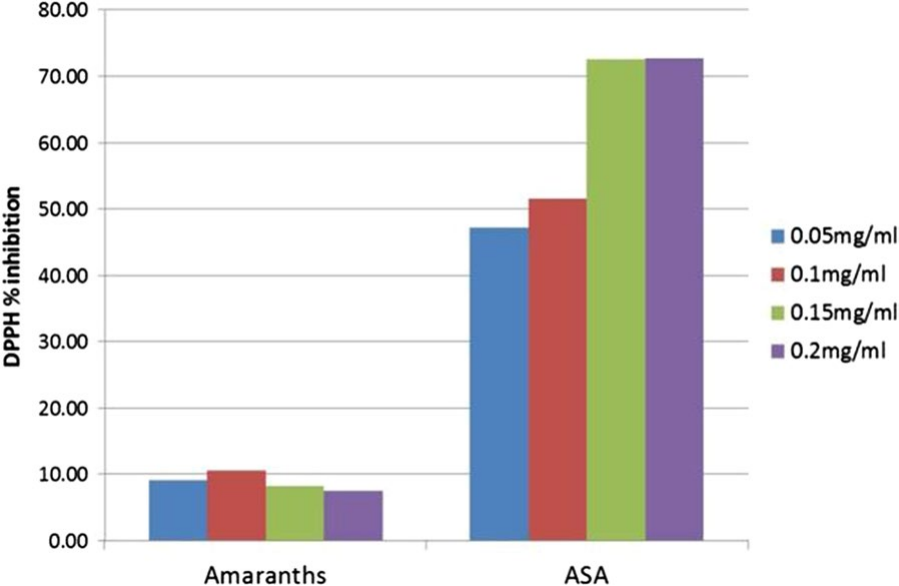
In vitro DPPH scavenging activity of *Amaranth* leaf extract

Amaranth leaves extract at concentration of 0.1 mg/ml had the highest (10.49%) inhibition of DPPH free radical as shown in Fig. 1.

**Fig. 2:**
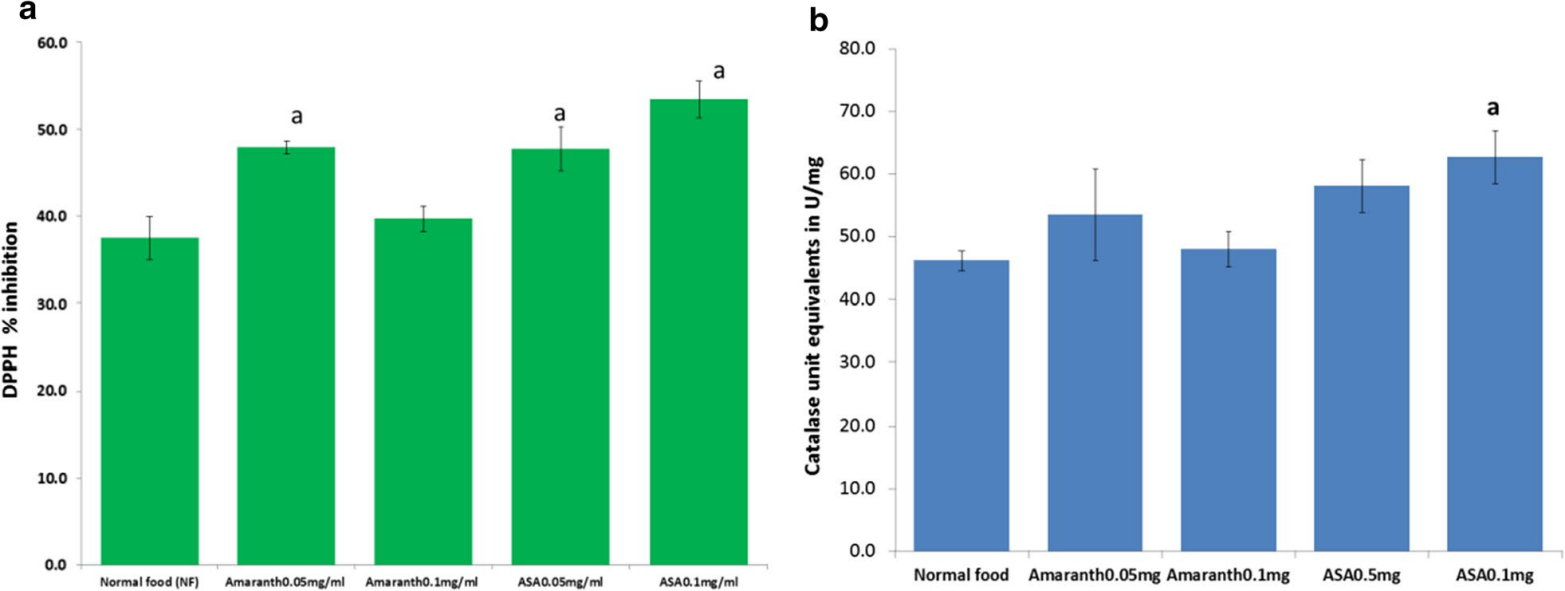
In vivo antioxidant activity of Amaranth leaf extract. **a** DPPH scavenging activity **b** Catalase enzyme activity

Homogenates from flies fed on food containing Amaranth or ascorbic acid at 0.05 mg/ml respectively had significantly higher DPPH scavenging activity compared to homogenates from flies fed on normal food as shown in Fig. 2. However, the increase was not significant at 0.1 mg/ml of Amaranth extract. The increase was similar to that observed when supplementing food with ascorbic acid. There was no significant increase in catalase activity in Drosophila flies fed on food supplemented with Amaranth leaf extract.

**Fig. 3:**
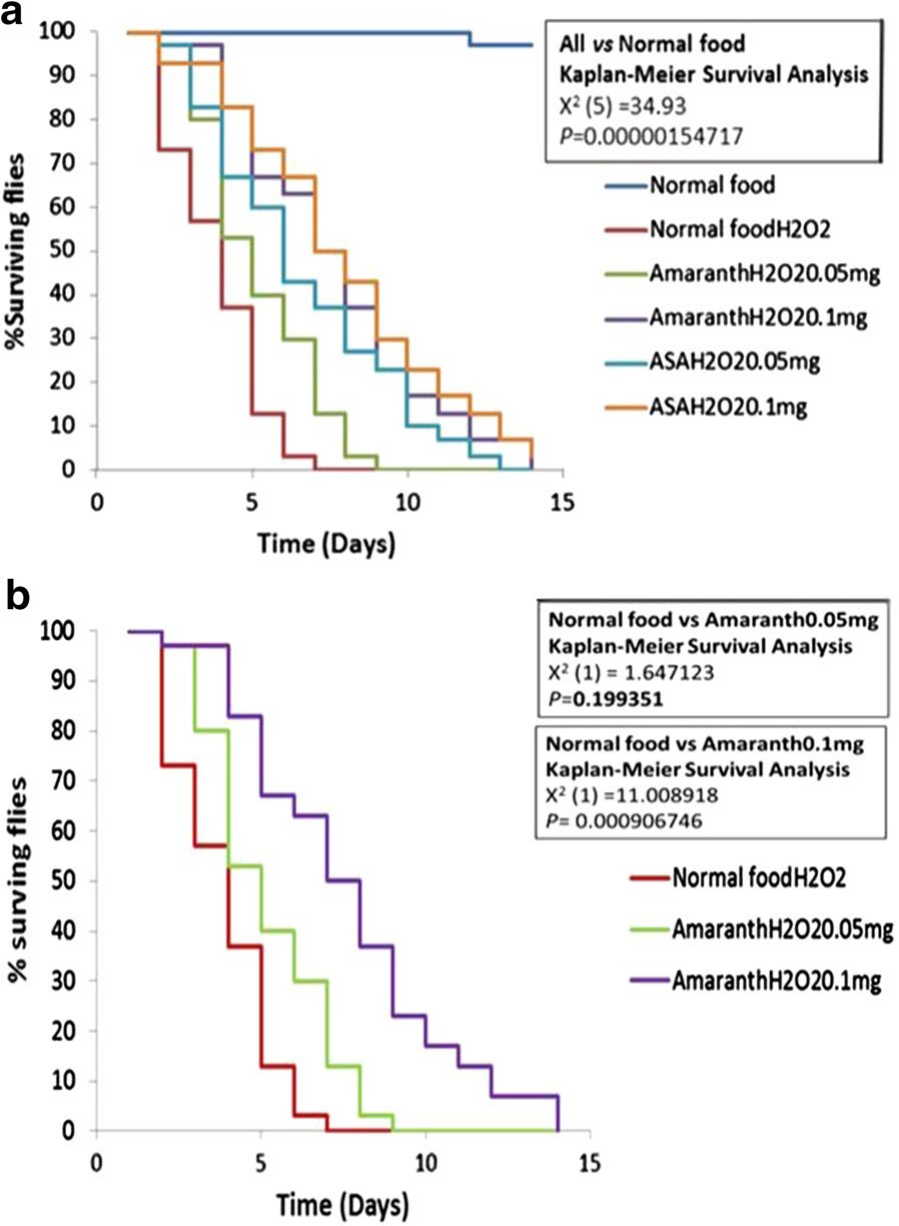
Survival of flies exposed to hydrogen peroxide. **a** All flies exposed to hydrogen peroxide showed reduced survival. **b** Supplementing food with Amaranths for 5 days improved survival rate

Supplementing food with Amaranth extract improved survival of flies exposed to H_2_o_2_ in a dose dependent manner. Flies fed on Amaranth extract at 0.05 mg and exposed to H_2_o_2_ survived longer (8 days) compared to those exposed to NF and H_2_o_2_ (6 days). This change was not statistically significant (p = 0.1993). However, there was a statistically significant (p = 0.0009) improvement of survival at 0.1 mg/ml of Amaranth extract which was similar to the protection offered by ascorbic acid Fig. 3.

## Discussion

Green leafy vegetables such as Amaranths contain antioxidant vitamins including ascorbic acid, α-tocopherol and β-carotene. However, most of their antioxidant activity is from polyphenol compounds such as flavonoids, isoflavones, flavones, anthocyanins, catechin and isocatechin [18–20]. Intake of compounds containing exogenous antioxidants is associated with an enhancement of endogenous antioxidant defenses [9, 15]. This has also been seen in this study with the increase in the in vivo DPPH free radical scavenging activity seen in *Drosophila* flies fed on Amaranth leaf extracts. Exogenous antioxidants can also modulate endogenous enzyme activity [5]. For example, catalase activity is increased in *Drosophila* flies fed on mango tree leaf extracts [15]. However, in this study the increase in catalase activity in flies fed on Amaranth extract was not significant.

Oxidative stress is an important factor in the pathogenesis of many diseases [4]. Several plant extracts with antioxidant activity have been shown to ameliorate effects of oxidative stress induced by compounds such as paraquat and hydrogen peroxide [10, 21–24]. The concentration of Amaranths that provides in vivo protection to oxidative stress does not significantly increase general in vivo antioxidant activity (DPPH scavenging activity) or the catalase enzyme activity. This protection, therefore, may be due to the interaction of the extract with other endogenous biochemical pathways or other compounds found in Amaranth leaves such as hyrolysates, bioactive peptides and 24, 25 dihydroxycholecalciferol [25, 26]] which further studies could address.

In conclusion, our study demonstrates that the ethanolic extract of Amaranth leaves offer protection against hydrogen peroxide-induced oxidative stress in vivo in a dose dependent manner.

## Limitations

These results are limited to male flies. Studies on female flies would provide more information on any gender related variations. Although Amaranths are widely consumed by humans, this study has been done in a lower invertebrate model and there is need for further testing in a vertebrate model before the results can be extrapolated to humans. The results are also limited to hydrogen peroxide induced oxidative stress and catalase enzyme. They cannot be used to draw conclusions on other antioxidant pathways.

## Supplementary Information

**Additional file1.** Supplementary file: Detailed Materials and Methods.

## Data Availability

The datasets used and/or analysed during the current study are available from the corresponding author on request.

## Abbreviations

H_2_o_2_: Hydrogen peroxide
ASA: Ascorbic acid
NF: Normal food
DPPH: 2, 2, diphenyl 1picrylhydrazyl
