# Supplementary material for: Amaranth leaf extract protects against hydrogen peroxide induced oxidative stress in Drosophila melanogaster
Source: BMC Res Notes. 2021 May 17;14:188. doi: 10.1186/s13104-021-05603-x (PMC8130338; doi:10.1186/s13104-021-05603-x)
Supplement: Supplementary file 1 — Additional file1. Supplementary file: Detailed Materials and Methods. [file 13104_2021_5603_MOESM1_ESM.pdf]

Amaranth leaf extract protects against hydrogen peroxide induced oxidative stress in *Drosophila melanogaster*

Authors

Ndinawe Johnmark[1] and Hellen W. Kinyi[2]*

Supplementary file 1: Methods

1. **Amaranth leaf extraction**

Amaranth leaves were air dried at room temperature for two weeks. Further drying was done in a dry oven at 30°C for 24 hours until constant weight was achieved. The dry leaves were then ground using mortar and pestle to powder form and sieved. Each 200 mg of ground material was cold extracted using 70% ethanol at a ratio of 1:10 respectively. The mixture was shaken and left to stand for 24 hours at room temperature. The mixture was then filtered using a Buckner Funnel and Whatman No 1 filter paper. Each ethanol filtrate was concentrated by evaporating the ethanol at 40°C over a water bath. Further drying was done in a hot air oven at 40^{0}C. The extract was collected and stored in small bottles at 4 °C. (1)

2. **In vitro DPPH Assay**

The DPPH radical-scavenging assay of Amaranth leaf extracts against stable DPPH (2,2-diphenyl-1-picrylhydrazyl radical) was determined spectrophotometrically by a slight modification of the method described in Stock solutions of crude extracts were prepared as 1 mg/ml in ethanol. 1ml of different concentration samples were added to 5ml of 0.004% methanol solution of DPPH. After 30 min of incubation in the dark at room temperature, the absorbance was read against a blank at 517 nm. (2)

The assay was carried out in triplicate and percentage of inhibition was calculated using the following formula:

$$\%\text{Inhibition} = ((AB-AA)/AB) \times 100$$

Where AB = Absorbance of blank; AA = Absorbance of sample.

3. Catalase enzyme activity assay

The assay was carried out on *Drosophila* flies fed on the NF, Amaranth and ASA food for five days. Ten flies per group anaesthetized by chilling on ice were homogenized in ice cold phosphate buffer saline (pH 7.4) at a weight: buffer ratio of 1:10 and centrifuged at 2,500rpm for 10 minutes. The resulting supernatant was collected and used for the catalase assay.

A standard curve was created using standard catalase at different concentrations using method described by (3). Catalase activity unit was defined as the number of micromoles of hydrogen peroxide decomposed per unit time.

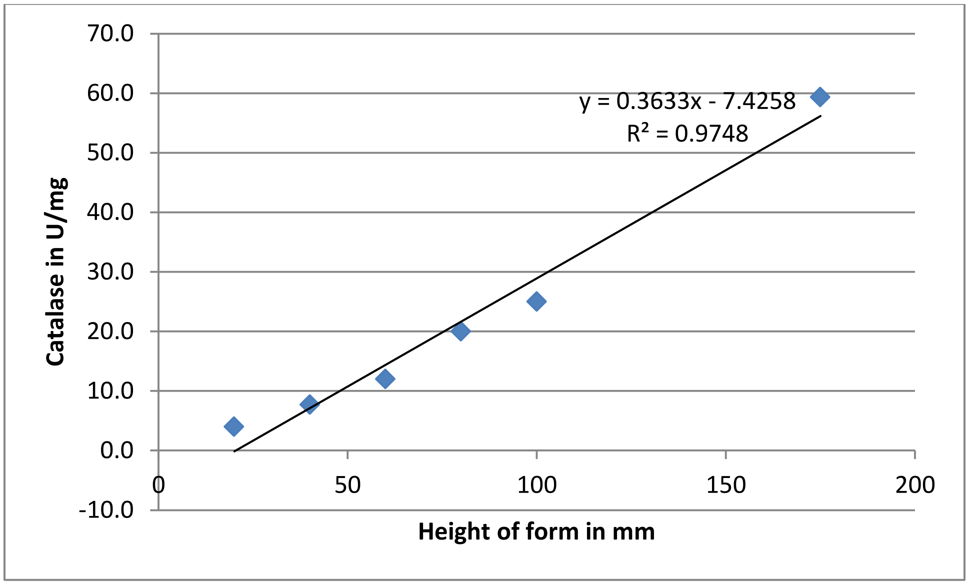

Figure 1: Standard curve of catalase activity

For the *in-vivo* catalase activity, 100 ml of fly homogenate solution was added to a Pyrex tube (13 mm diameter × 100 mm height, (Borosilicate glass; Corning, USA). Subsequently, 100 ml of 1% Triton X-100 and 100 ml of undiluted hydrogen peroxide (30%) were added to the solutions and mixed thoroughly and were then incubated at room temperature. Following completion of the reaction, the height of O2-forming foam in the test tube that remained constant for 15 minutes was measured using a ruler and catalase activity deduced from the standard curve.

References

1. Maina E, Kinyi HW, Ochwangi D, Meroka A, Wanyonyi W. Liver toxicity of Crude extract of Ficus natalensis traditionally used in South Western Uganda. African J Pharmacol Ther [Internet]. KE; 2013 Dec 31;3(4):116–21. Available from: http://journals.uonbi.ac.ke/ajpt/article/view/1257

2. Samarth RM, Panwar M, Kumar M, Soni A, Kumar M, Kumar A. Evaluation of antioxidant and radical-scavenging activities of certain radioprotective plant extracts. Food Chem. 2008;106(2):868–73.

3. Iwase T, Tajima A, Sugimoto S, Okuda K, Hironaka I, Kamata Y. A Simple Assay for Measuring Catalase Activity : A Visual Approach. 2013;3–6.
